# *Eucalyptus* research in the post-genome era

**DOI:** 10.1186/1753-6561-5-S7-I22

**Published:** 2011-09-13

**Authors:** Georgios Pappas, Sergio de Alencar, Orzenil B Silva-Junior, Roberto C Togawa, Marilia CR Pappas, Dario Grattapaglia

**Affiliations:** 1 EMBRAPA Genetic Resources and Biotechnology - EPqB FInal W5 Norte, Brazilia DF 70770-917, Brazil

## 

With the efforts of the Joint Genome Institute (JGI) through the EUCAGEN Eucalyptus Genome Network reaching the final release of a *Eucalyptus grandis* reference genome (BRASUZ1), it is anticipated that this accomplishment will profoundly shape the future research of this global tree genus [1]. One of the first steps toward this end has been the refinement of the genome annotation. Robust models have been generated for protein-coding genes. Here we report the annotation of other pivotal genome constituents, transposable elements (TE) and micro RNA genes. TEs are not only the most dominant elements but are also major drivers of genome plasticity. Several bioinformatic strategies were employed to perform “de novo” and homology based prediction of repetitive elements [2]in the current genome release (version 1.0). A total of 53 distinct TE families could be identified, with the retrotransposon super-family being widely over-represented, as observed for the majority of plant genomes sequenced.

Micro RNAs (miRNA), key players in post-transcriptional gene regulation, were annotated using a combination of massively parallel sequencing of small RNA libraries and a genome-wide computational screening to ascertain a compatible secondary structure of the precursor. Both experimental and “in silico” evidences enabled the annotation of 206 distinct miRNA loci comprising 36 different mir gene families including several miRNA isoforms. The blueprint provided by a high-quality reference genome, both in terms of sequence completeness and annotation, will leverage efforts to better characterize intra- and inter-specific sequence variation underlying the marked phenotypic differences among the hundreds of species comprising this genus.

Recent advances of DNA sequencing technologies permit a comprehensive interrogation of several other individuals at a fraction of the cost. In this context, we have used Illumina (2x75bp) short read sequencing data of *E. globulus* clone X46 generated by JGI and made available through the EUCAGEN network to carry out two comparative genomics experiments. From 40X raw sequence data provided it was possible to use an equivalent of 20X coverage (~12Gbp). We first attempted to perform a "de novo" assembly using VELVET [3]. A total of 161,000 contigs were obtained the largest one sizing at ~3,5kb. In spite of the easy access and low cost of next generation sequencing technologies, these results suggest that even for relatively small forest tree genomes, current technical and computational limitations preclude comprehensive assembly, likely due to the ubiquitous occurrence of repetitive elements in such genomes. Nevertheless when we mapped the *E. globulus* sequencing data against the BRASUZ1 reference genome, 55% of the reads could be mapped with high confidence. From these, approximately 800,000 high quality single nucleotide polymorphisms (SNPs) could be identified clearly showing the key role that the reference genome will have for future genomic undertakings. The sheer number of molecular markers discovered in this experiment not only fosters more powerful studies on the evolutionary history and population genomics of eucalypts, but also inaugurates a new era in molecular breeding of species of this genus, providing genome-wide coverage for genomic selection and association studies.

## References

[B1] Grattapaglia D Kirst M Eucalyptus applied genomics: from gene sequences to breeding tools New Phytologist 2008 179 4 911 929 10.1111/j.1469-8137.2008.02503.x 18537893

[B2] Flutre T Duprat E Feuillet C Quesneville H Considering Transposable Element Diversification in De Novo Annotation Approaches Plos One 2011 6 1 10.1371/journal.pone.0016526 PMC3031573 21304975

[B3] Zerbino DR Birney E Velvet: Algorithms for de novo short read assembly using de Bruijn graphs Genome Research 2008 18 5 821 829 10.1101/gr.074492.107 18349386 PMC2336801

